# Development of a theory-based questionnaire to assess structure and control in parent feeding (SCPF)

**DOI:** 10.1186/s12966-017-0466-2

**Published:** 2017-01-26

**Authors:** Jennifer S. Savage, Brandi Y. Rollins, Kari C. Kugler, Leann L. Birch, Michele E. Marini

**Affiliations:** 1 Center for Childhood Obesity Research, The Pennsylvania State University, University Park, PA 16802 USA; 2 Department of Nutritional Sciences, The Pennsylvania State University, University Park, PA 16802 USA; 3 The Methodology Center, The Pennsylvania State University, 204 E. Calder Way, Suite 400, University Park, PA 16801 USA; 4 Department of Foods and Nutrition, University of Georgia, 176 Dawson Hall, Athens, GA 30602 USA; 5 Department of Nutritional Sciences, Center for Childhood Obesity Research, The Pennsylvania State University, 129 Noll Laboratory, University Park, PA 16802 USA

**Keywords:** Feeding practices, Scale development, Parenting, Theoretical, Toddlers aged 12 to 36 months, Low-income, Structure, Control, Exploratory factor analysis

## Abstract

**Background:**

Parents shape children’s eating environments and act as powerful socialization agents, impacting young children’s behavioral controls of food intake. Most feeding measures assess parents’ use of control to manage children’s intake of energy dense foods. The Structure and Control in Parent Feeding (SCPF) questionnaire was developed to assess more positive aspects of feeding practices with their young children —setting limits, providing routines—that promote self-regulation, as well as controlling feeding practices.

**Methods:**

A mixed method approach was used to develop the SCPF. In 2013, cognitive interviews informed the modification, deletion and/or replacement of items. In 2014, the survey was distributed statewide to mothers of toddlers aged 12 to 36 months participating in the Women, Infants, and Children program. In 2016, exploratory factor analyses was conducted to test our theoretical parenting model and content validity and criterion validity were assessed (*n* = 334).

**Results:**

Exploratory factor analysis (EFA) and second-order EFA revealed a 2-factor, 22-item Structure model and a 2-factor, 12-item Control model. Internal consistencies for all factors exceeded 0.70. As predicted, the Structure superfactor was positivity associated with responsiveness, whereas the Control superfactor was positively associated with demandingness on the Caregiver’s Feeding Styles Questionnaire. The Structure subscales were also positively associated with mealtime behaviors and Control subscales were positively associated with control-oriented feeding measures from the Control in Parent Feeding Practices questionnaire.

**Conclusion:**

The SCPF questionnaire is a reliable tool that can be used to assess aspects of structure- and control-based feeding practices to better understand how parents feed their toddlers.

## Background

Obesity risk among infants and toddlers continues to remain a significant public health concern [1], particularly among children of low-income families who are disproportionately affected [2, 3]. The transition from infancy to toddlerhood has been identified as a critical period for establishing dietary intake patterns, eating habits, and food preferences [4]. It is also a time when parents are powerful socialization agents, structuring children’s eating environments, thereby shaping children’s developing behavioral controls of food intake that serves as the foundation for future eating behaviors, intake patterns and obesity risk [5–7]. Parents act as the gatekeepers to food and beverages, setting limits, establishing routines or imposing more coercive control over what, when, and how much young children eat and drink, and how food and drink is served [8]. Identifying parental characteristics that affect the development of children’s eating behavior, self-regulation, and obesity risk can provide the evidence base for primary prevention of childhood obesity.

Most of the discussion on parent feeding practices has focused solely on aspects of parental feeding that are viewed as problematic - controlling, intrusive or coercive practices to control what and how much children. Controlling feeding practices such as pressuring children to eat or restricting access to palatable foods have counterproductive effects on the development of children’s self-regulatory skills affecting food intake [9, 10] and weight status. These parenting practices provide few opportunities for children to make choices about food and eating and to develop self-regulatory skills. In the case of restriction, children may actually learn to like and eat more of the restricted food [9, 10]. Although this evidence can inform guidance for parents about what *NOT* to do, parents are often left asking “*then what should I do?*” to manage children’s intake of palatable, energy-dense foods within our current obesigenic environment. Potential answers to this question are suggested from the work of Grolnick and Pomerantz [11]. In this paper, we describe the development and initial validation of the Structure and Control in Parent Feeding (SCPF) questionnaire using Grolnick and Pomerantz’s theory of parental control [11].

Grolnick and Pomerantz’s [11] model of parental control provides insight into how parental control in the feeding domain can be conceptualized and measured [12]. In this model of parenting, two qualitatively different independent factors (e.g., structure and control) are posited to differentially influence the development of children’s self-regulatory skills. Parental control is characterized by coercion, coldness, and intrusive parenting behaviors that can minimize opportunities for children to practice autonomy, and develop and practice self-regulatory skills. Grolnick and Pomerantz also argue that controlling parenting *is not* structure-based parenting. Structure is defined as non-coercive [13] communication of clear, consistent guidance and expectations that considers the child’s perspective, and provides children with limits while still providing opportunities to practice and develop self-regulatory skills [11]. In other words, control is coercive and intrusive while structure provides clear limits and routines.

Intrusive, controlling parenting negatively impacts children’s development of self-regulation, these practices are associated with greater eating in the absence of hunger and reduced ability to delay of gratification in children [14, 15], and higher weight status in adulthood [16] In contrast, structure-based, limit setting practices have been shown to promote child social and emotional regulation [11, 15]. A lack of structure or guidance in parenting can be detrimental to child development of self-regulation skills [17, 18]. Thus, setting clear and consistent limits and routines around eating that provides structure and predictability to the current food environment, while still allowing the child some degree of autonomy within those constraints, can foster children’s self-regulatory skills and reduce consumption of palatable energy-dense foods [19–22]. This could then in turn support patterns of intake consistent with healthy growth and development [23–25]. Using Grolnick and Pomerantz’s theory, examples of structure-based parent feeding practices would include setting limits around what, when and how much food is available, providing routines and rules around eating and mealtimes, consistently implementing these rules, and limiting children’s exposure to unhealthy foods.

Despite limited evidence that parents’ use of structure-based feeding may positively impact children’s food intake by reducing their consumption of energy dense foods [19, 20, 22], most parent feeding surveys are limited to assessing control based feeding practices. In a 2013 review [26] including over 70 parent feeding measures, control and structure-based feeding practices have not been conceptualized and measured as separate constructs. In addition, very few surveys have been designed specifically for toddlers, the period between 12 and 36 months when children are becoming more independent, are developing the requisite cognitive and communication skills to support the development of self-regulatory skills, including inhibitory control, delay of gratification, and emotion regulation; all skills essential to healthy development, including healthy eating and weight status. Taken together, there is a need for a feeding measure that provides a clear operational definition of what control in feeding *is* (e.g. restriction, pressure) and what *is not* control (e.g. setting limits, routines) *and* considers parents’ need to manage children’s intake of energy-dense foods within our obesigenic environment.

The goal of this research is to develop and validate a new self-report measure of toddler feeding practices that is designed to include dimensions of control in feeding and structure in feeding, based on Grolnick and Pomerantz’s [11] conceptual model from the parenting literature that provides the framework for item generation. This measure was intended for use among a sample at relatively high risk for obesity: low income mothers of toddlers (age 12- to 36 months) participating in the Special Supplemental Nutrition Program for Women, Infants, and Children (WIC).

## Methods

A mixed method approach was used to develop the Structure and Control in Parent Feeding (SCPF) questionnaire comprising three steps: (1) items were identified from existing parenting questionnaires and developed based on the Grolnick and Pomerantz framework; (2) cognitive interviews with mothers of toddlers informed the modification, deletion and/or replacement of items; and (3) Exploratory factor analysis (EFA) and second-order factor analysis were conducted to test our theoretical parenting model of control and structure, and content validity and criterion validity were assessed.

### Step 1: item generation

A literature review was conducted to identify survey instruments assessing dimensions of parent feeding practices and general parenting of infants, toddlers, and preschool aged children. An item bank was created from the following list of instruments (details can be found in Table 1): Comprehensive Feeding Practice Questionnaire [27], Child Feed Questionnaire [28], feeding style items developed by Hurley et al. [29], Infant Feeding Questionnaire [30], Infant Feeding Styles Questionnaire [31], Control in Parent Feeding Practices questionnaire [32], and items from a published dissertation that included a measure titled the Toddler Feeding Questionnaire [33]. Our research team held meetings to review the items, determine face validity of each item, and to make modifications to items that were awkwardly worded or that needed to be adapted for toddler-aged children. The authors also developed 17 additional items to capture dimensions of controlling and structure-based feeding, informed by the general parenting literature: 4 items related to restriction, 3 items related to food as reward, 3 item related to limit-setting and 3 items related to signs of fullness.

**Table 1:** Structure and control based feeding practice listed by feeding items^a^ with their factor loadings. (*N* = 334)

				Structure-based feeding practice		Control-based feeding practice	
#	Items^a^	Item Source	Item response (%)^b^	Limit Exposure	Consistent Feeding Routines	Restriction	Pressure to Eat
8^c^	Keep a lot of snack foods (potato chips, cheese puffs, tortilla chips) in my house	CFPQ (Environment)	57.4	*.55*	.02	-.01	-.14
28^c^	Keep a lot of sweets/desserts (candy, ice cream, cake, pies, pastries) in my home	CFPQ (Environment)	71.6	*.62*	.08	-.04	-.19
4^c^	Serve child sweets or desserts (ex. Cookie, cake, candy, freeze pops, ice cream)	IFSQ^e^ (Permissive)	40.8	*.59*	.07	.07	.01
6 ^c^	Serve child sugar sweetened drinks (ex. fruit drink, soda, iced tea, or sports/energy drinks)	IFSQ^e^ (Permissive)	65.5	*.58*	.26	.03	.00
14 ^c^	Serve child French fries.	Author developed	31.3	*.45*	.18	.16	-.02
40 ^c^	Let child have sugar sweetened drinks (ex. Fruit drink, soda, iced tea, or sports/energy drinks) anytime during day	Hurley^e^ (Indulgent)	77 .9	*.58*	.33	.11	-.09
2 ^c^	Let child eat sweets (ex. Cookie, cake, candy, freeze pops, ice cream) anytime during the day	Author developed	63.1	*.53*	.28	.14	-.11
1	Avoid buying sweets/desserts I don’t want child to eat	Author developed	59.2	*.45*	.40	.34	-.05
43	Try not to eat unhealthy foods when child is around	CFPQ^e^ (Modeling; revised from eating healthy to avoiding unhealthy foods)	55.6	*.42*	.28	.37	.10
3	Avoid eating snacks/sweets in front of child that you don’t want him to eat	CPFP^e^ (energy dense food encountering practice)	65.0	*.46*	.35	.36	-.17
41	I do not allow other people to give sweets and snacks to my toddler without asking me	TFQ^e^	63.4	*.43*	.26	.20	.18
21^c^	Let child eat snack foods anytime during the day^d^	IFSQ^e^ (Laissez-faire)	70.8	.36	*.36*	.21	-.07
30	Serve child green/yellow/orange vegetable each day	Author developed	74.2	.24	*.43*	.08	.13
37	Child eats breakfast at same time and place	Author developed	87.0	.15	*.64*	.14	-.03
36	Serve small child size helpings at meals	CFPQ^e^ (restriction for weight control; removed “due to weight”	80.2	.21	*.47*	.22	-.01
39	Child eats dinner at same time each night (within about 15 min)	Author developed	73.8	.22	*.59*	.10	-.02
26	Keep a lot of fresh fruits/vegetables in my home	CPFP^e^ (removed; didn’t load on original scale)	89.3	.20	*.44*	.12	.14
7	Serve small child size helpings at snacks	CFPQ^e^ (restriction for weight control; changed meal to snack and removed “due to weight”	79.2	.05	*.44*	.08	-.11
19	Have child sit in a chair when eating meals	CPFP^e^ (Mealtime behavior)	88.5	.09	*.43*	.07	-.19
5	Have clear rules about when child can eat snacks/sweets	TFQ^e^	69.5	.34	*.45*	.38	.09
35	Child eats at scheduled meal and snack times, not in-between	CFPQ (Restriction for weight control, deleted “because I don’t want him/her to get fat)	41.8	.21	*.44*	.29	.15
34	I have very firm rules about what types of foods I allow my toddler to have (with no exceptions)	TFQ	46.5	.23	*.41*	.38	.25
9	I hide foods that I don’t want my child to eat	CFQ^e^ (restriction)	40.1	.12	.16	*.75*	.09
10	If my child is eating too much, I take some of it away	CFQ^e^	26.0	-.09	.12	*.45*	.18
18	When my child is drinking too much of a sugar-sweetened beverage, I take the cup/bottle away or pour some out	Author developed	46.7	.04	.17	*.52*	.17
42	In my home, I hide snack foods from my child that I don’t want my child to eat	CFQ/TFQ^e^ (restriction/ access)	28.0	.17	.17	*.71*	.20
13	I get upset when my child eats too many snacks or salty foods without asking	Author developed	35.5	.08	.07	*.54*	.18
11	Avoid taking child to places where my child may ask for sweets, snacks, junk	TFQ	14.8	.15	.10	*.62*	.18
38	I have to trick, distract, play with, or praise my child to get him/her to finish his/her bottle/food.	Hurley (Forceful)	7.6	-.13	-.21	.21	*.52*
23	If I did not control my child’s eating, he/she would eat much less than he/she should	CFQ^e^	17.5	-.09	-.10	.19	*.55*
27	I try to get my child to finish his/her food.	Hurley (forceful)	51.7	.03	.15	.11	*.65*
24	If my child seems full, I encourage him/her to finish his/her food anyway.	IFSQ^e^ (responsive)	8.4	-.03	-.08	.14	*.54*
33	I try to get my child to eat even if s/he doesn’t seem hungry or says “I’m not hungry”	CFQ^e^	14.7	-.14	-.06	.11	*.53*
25	I praise my child after each bite to encourage him/her to finish his/her food.	IFSQ^e^ (pressure)	41.3	-.05	.14	.21	*.53*
Other practices included in EFA, but eliminated because items had loadings less than .40							
22	Child drinks milk at dinner every night	Author developed	45.6				
16	Let child eat directly out of regular-sized snack bags (ex. Bags of chips/pretzels, box of cookies, candy, etc.)	Author developed	83.9				
31	Have all of child’s favorite foods at home	Author developed	7.5				
20	At mealtimes, I give child same amount of foods as I serve myself	Author developed	89.7				
17	Give child same amount of snacks as I serve myself	Author developed	89.7				
29	I like to have complete control over what types of sweets and snacks my toddler is able to eat (is given)	TFQ	78.0				
46	I offer food to get my child to do what I want him/her to do	Author developed	0.0				
44	I offer my child his/her favorite foods as a reward for good behavior	CFQ/CFPQ (restriction/ food as reward)	6.6				
45	I withhold sweets/desserts from my child in response to bad behavior	CFQ/CFPQ (restriction/ food as reward)	11.5				
47	I offer my child a “treat” or “dessert” for eating everything on his/her plate	Author developed	13.8				
50	I offer my child a “treat” or “dessert” to get my child to eat his/her vegetables	Author developed	5.1				
48^c^	If I’ve told child “No, you can’t have it,” likely to give it to him anyway	TFQ^e^	82.9				
49^c^	Likely to give child whatever he wants to eat.	Hurley^e^ (Indulgent)	52.2				
12^c^	Let child watch TV while eating	IFSQ^e^/CPFQ^e^ (Indulgent/ mealtime behavior)	55.4				
32	If child doesn’t like what we are eating, I fix something else for child to eat	IFQ^e^	41.5				
15	Child eats with me/another caregiver at table each night	Author Developed	90.0				

^a^Scored on a 5-point likert scale: 0 = never and 4 = always

^b^.Percentage of responses between 2 (sometimes) and 4 (always)

^c^Reverse coded

^d^ Factor membership is determined if an item loaded at 0.40 or greater with one exception. Item 21 was retained in analysis despite loading <0.4 because it was consistent with “Feeding Consistent Routines” factor

^e^Item was modified from the original scale following cognitive interviews

*Abbreviations*: *CFPQ* Comprehensive Feeding Practice Questionnaire [27], *CFQ* Child Feed Questionnaire [28], *Hurley* Feeding style items developed by Hurley et al. [29], *IFQ* Infant Feeding Questionnaire [30], *IFSQ* Infant Feeding Styles Questionnaire [31], and *CPFP* Control in Parent Feeding Practices questionnaire [32], Toddler Feeding Questionnaire [33]

### Step 2: cognitive interviews

Project staff recruited 70 mothers from two WIC clinics in central Pennsylvania; eligibility criteria included having a toddler, defined as between age 12 and 36 months. Mothers were asked to complete a survey about feeding their toddler, which consisted of the items selected in the item generation phase. After they completed the survey, research staff read the items aloud to the participant to determine whether mothers understood the phrasing of each item as intended. Mothers identified items that they were unable to answer, that were not clear, had multiple meanings, or were too complex. Based on the results of the interviews, items were selected; several items were revised and one item was removed due to low face validity, resulting in a pool of 50 items.

### Step 3: test higher-order two-factor model of control and structure and assess validity of the SCPF

#### Participants and data collection

A total of 550 surveys were distributed statewide to 18 Pennsylvania WIC clinic directors, with 200 surveys allocated to urban clinics and 350 surveys to rural clinics. WIC clinic staff recruited and consented mothers who met the eligibility criteria: English speaking mothers who were 18 years or older with a child aged 12 to 36 months. WIC clinic staff instructed participants to complete a demographic questionnaire and the SCPF questionnaire (see description below) on site. Participants also completed a longer survey at home that included items used to assess attitudes and norms towards feeding practices, parenting practices, and parent feeding styles. Of the 550 on-site surveys that were distributed to WIC clinics to administer, a total of 431 were completed and returned by participants who met the eligibility criteria; 71 surveys were completed by women with children who did not meet child age eligibility. Of these 431 surveys, a total of 334 participants also completed the longer, take-home survey. Participants earned up to $35 in gift cards for completing the surveys. To encourage clinic staff to assist with recruitment efforts, participating clinics were entered into a drawing to receive a catered lunch valued up to $200. All procedures and measures were approved by Penn State’s IRB.

#### Measures

To assess criterion validity of the SCPF questionnaire, we administered the Control in Parent Feeding Practices (CPFP) questionnaire and the Caregiver’s Feeding Styles Questionnaire (CFSQ) as part of the longer, take-home survey. The CPFP questionnaire [32], a 24-item instrument that assesses parent reported directive, non-directive, and environmental control in feeding, has acceptable reliability and model fit among preschool aged children [32]. In the current study, internal consistency coefficients were good: child-centered feeding (α = .75), nutrient-dense food encouraging practices (α = .62), energy-dense food discouraging practices (α = .65), mealtime behavior (α = .58), timing of meal (α = .67), parent high control included 3 items (α = .65), and parent high contingency (α = .82). The CFSQ is a parent-report questionnaire that distinguish patterns of child food parenting: parental demandingness and parental responsiveness [34]. Parental demandingness included all 19 items (α = .88) and parental responsiveness included the mean of 7 child-centered items divided by the total mean of the 19 items (α = .75). Per author recommendations, median splits were used to dichotomize participant responses into high and low categories on demandingness and responsiveness to create four parenting typologies: authoritarian, authoritative, indulgent, and uninvolved.

Additional measures included maternal socio-demographic characteristics such as age, marital status, race/ethnicity, employment, education, number and description of people in the household, and maternal obesity status using self-reported data (obese defined as BMI ≥ 30 versus not obese). Depressive symptom status was assessed using the Center for Epidemiologic Studies of Depression Scale (CES-D) [35]; with risk of depressive symptomology being scored as CESD score ≥ 16. Child characteristics included child’s age (in months).

#### Statistical analyses

Analyses were conducted using data from a final sample of 334 participants who met eligibility criteria. Modern missing data approaches (regression imputation using maximum likelihood estimations in AMOS/SPSS software) was used to impute the missing data for all our analyses which enabled us to maintain the power associated with a full sample. The theorized factor structure of the proposed 50 SCPF items was investigated using the following process. First, an EFA (SAS, PROC FACTOR) using an iterated principal axes factor extraction with an oblimin rotation was performed on all 50 items to identify the first-order factor structure of the data. The following were examined to select the best fitting model: loading values, the scree plot, eigenvalues (>1), and the interpretability of the factor solution. Items that did not achieve a factor loading of 0.4 or greater were individually removed, the model retested, and the above repeated until all remaining factors loaded at 0.4 and no items loaded on more than one factor [36]. To perform the second-order factor analysis, the correlation matrix from the above EFA was generated and outputted and read into a second Proc FACTOR, using the same extraction and rotation above, but this time using only the correlation output (*n* = 334) (original sample size) [37]. Psychometrics for the SCPF questionnaire were evaluated by 1) computing internal reliability for the superfactors and subscales, and 2) Pearson correlations for convergent/divergent validity between the SCPF scales and the subscales from the CFSQ and CPFP measures. In addition, ANOVA was run to determine how the SCPF scales were related to the parenting styles. For internal consistency, we used the criterion of an alpha > .70 as desirable [38].

## Results

Mothers were on average age 29 (+7) years, 40% were married, 43% were unemployed, 26% were employed full-time, and the majority were Caucasian (72%), followed by African American (16%) and mixed race/other (12%). Half of mothers reported having some college education, followed by 37% who completed high school, and 13% with less than a high school education. Almost half of the mothers were overweight or obese (42%). Lastly, 29% reported depressive symptomology (CESD score > 16). Toddlers were on average age 22 (+7) months.

### Exploratory factor analysis (EFA) and second-order exploratory factor analysis

An EFA with a direct-oblimin rotation of the 50 items with an iterative principal axes factors extraction identified 5 factors with eigenvalues exceeding 1.0, and explained 75% of the variance. The ‘elbow’ in the scree plot suggested between 4 and 5 factors. The initial EFA with 5 factors produced 1 factor with only 2 items, and so a four-factor model was selected based on interpretability, still explaining 75% of the variance. Sixteen items were removed because they had loadings less than .40. One item (“*Let child eat snack foods anytime during the day*”) that produced a loading of .36 was maintained in the model because it was consistent with the items within a factor related to feeding consistency. After the previously identified items were eliminated, no items loaded on more than one factor. The final model included 34 items loading on four factors: Factor 1: Limit Exposure (11 items), Factor 2: Consistent Feeding Routines (11 items), Factor 3: Restriction (6 items), and Factor 4: Pressure to Eat (6 items) and the item loadings are presented in Table 1. Next, the correlation structure from the 4-factor model was used to further examine a second-order exploratory factor analysis to understand relations among the factors. Using the same EFA protocol (method = iterative principal factor axes extraction and direct oblimin rotation) indicated that Factors 1 and 2 loaded onto a single superfactor (factor loadings for superfactor 1 = 0.54 and 0.59, respectively and factor loadings for superfactor 2 = −0.01 and 0.12, respectively) and Factors 3 and 4 loaded onto a separate superfactor (factor loadings for superfactor 2 = 0.53 and 0.51, respectively and factor loadings for superfactor 1 = 0.39 and −0.05, respectively). Based on the nature of the items, and the way in which the items load as a unit, we identify the first of these 2 superfactors as “Structure” and the second as “Control”.

### Internal consistency

Summary descriptives and Cronbach alphas for the structure-based and control-based parent feeding factors are presented in Table 2. Overall, the mean scores for the Structure subscales and superfactor were relatively higher than the mean scores for the Control subscales and superfactor. Cronbach alphas for all subscales and superfactors exceeded 0.70, with three of the four scales and both superfactors exceeding 0.75.

**Table 2:** Descriptive statistics and Cronbach alphas of the Structure-Control in Parent Feeding Questionnaire

	*M*	SD	Range^a^		*α*
			Min	Max	
Structure factors	2.9	0.4	1.5	3.8	.84
Limit exposure	2.7	0.5	0.3	3.9	.79
Mealtime routines	3.0	0.5	1.4	4.0	.75
Control factors	1.6	0.7	0	3.5	.76
Restriction	1.7	0.9	0	4.0	.77
Pressure to eat	1.6	0.7	0	4.0	.73

^a^Scored on a 5-point Likert scale: 0 = never and 4 = always

### Convergent validity

To determine convergent validity of the SCPF questionnaire, the SCPF superfactors and subscales were correlated with the subscales from the CFSQ and CPFP questionnaires (Table 3). As expected, the Structure superfactor and subscales were positively associated with responsiveness from the CFSQ and the less controlling, more structure-oriented subscales from the CPFP questionnaire, including discouraging energy-dense foods, encouraging nutrient-dense foods, mealtime behavior, and timing of meals. Among the Structure subscales, Limit Exposure was most correlated with discouraging energy-dense foods in the CPFP; Consistent Feeding Routines was most positively associated with CPFP mealtime behaviors.

**Table 3:** Relationships among the SCPF Structure and Control superfactor and subscales and maternal feeding practice that establish criterion validity from the Caregiver’s Feeding Styles Questionnaire (CFSQ) and the Control in Parent Feeding Practices Questionnaire (CPFP)

	Structure			Control		
	Limit exposure	Consistent feeding routines	Structure Superfactor	Restriction	Pressure to eat	Control Superfactor
CPFP feeding practices						
Child-centered feeding	0.02	0.12	0.07	0.20**	0.25***	0.27***
Nutrient-dense food encouraging practices	0.15*	0.28***	0.24***	0.05	0.04	0.06
Energy-dense food discouraging practices	0.37***	0.28***	0.39***	0.13*	−0.02	0.08
Mealtime behavior	0.30***	0.39***	0.40***	−0.02	−0.11	−0.08
Timing of meal	0.33***	0.19**	0.32***	−0.01	−0.15*	−0.09
High control	−0.17**	−0.18**	−0.20**	0.11	0.36***	0.27***
High contingency	−0.17**	−0.04	−0.14*	0.30***	0.36***	0.41***
CFSQ feeding practices						
Demandingness	−0.05	0.01	−0.03	0.28***	0.41***	0.42***
Responsiveness	0.22**	0.29***	0.29**	−0.15*	−0.20**	−0.22**
SCPF Questionnaire						
Restriction	0.22***	0.30***	.30***			
Pressure to eat	−0.09	0.06**	-.0.03			

*Abbreviations*: *SCPF* Structure and Control in Parent Feeding questionnaire, *CPFP* Control in Parent Feeding Practices questionnaire, *CFSQ* Caregiver’s Feeding Styles Questionnaire

*p < 0.05; **p < 0.01; ***p < 0.001

As shown in Table 3, the Control superfactor and its subscales were most positively correlated with the control-oriented subscales. Mother’s scores on the Control superfactor was associated with higher scores on demandingness and high controlling and high contingency. The Restriction subscale was most correlated with high contingency; the Pressure to Eat subscale was most correlated with high controlling and high contingency.

### Discriminant validity

Based on our theoretical framework, we expected that the Structure subscales would show either null or inverse associations with control-oriented feeding measures from the CFSQ and CPFP questionnaire. As expected and shown in Table 3, the Structure superfactor and subscales produced either negative or null associations with the control-oriented subscales: high controlling and high contingency and demandingness. We also expected inverse associations between the Control superfactor and subscales with more structure-based, responsive feeding subscales from the CFSQ and CPFP questionnaire. The majority of correlations were null or negative correlations ranging from -.08 to -.22. However, child-centered feeding was correlated with the Control superfactor and both of the Control subscales (correlations ranged from .20 to .27); and between the Control superfactor and nutrient-dense food encouraging practices.

Pearson’s correlations among Structure and Control subscales are also in Table 3. Pressure to Eat had either null or inverse correlations with the two Structure subscales and the Structure superfactor. However, Restriction was positively correlated with both Structure subscales, as well as the overall Structure superfactor, indicating that mothers who used more restrictive feeding practices tended to use more structure-based feeding. Lastly, the superfactors for Structure and Control were very weakly correlated (*r* = .20, *p* < .001), sharing less than 4% of their variance, suggesting that the superfactors are orthogonal.

### The SCPF questionnaire and parenting feeding styles

Maternal reports of Structure- and Control-based parent feeding factors and superfactors were evaluated by parental feeding styles, as measured by the CFSQ (Table 4). The most prevalent feeding style was indulgent, comprising 34.1% of our sample, followed by authoritarian (30.6%), uninvolved (19.2%), and authoritative (15.8%). As expected, authoritative and indulgent mothers tended to score the highest on the Structure superfactor and subscales, while uninvolved and authoritarian mothers had the lowest scores. In contrast, authoritarian mothers tended to have higher scores on the Control superfactor and subscales compared to authoritative mothers, however. Lastly, as expected, indulgent and uninvolved tended to have the lowest scores on the Control superfactor and subscales.

**Table 4:** Mean differences in structure and control feeding practices by parental feeding styles, as determined by the Caregiver’s Feeding Styles Questionnaire (CFSQ, *N* = 245)

	Authoritarian (*n* = 72)	Authoritative (*n* = 40)	Indulgent (*n* = 86)	Uninvolved (*n* = 47)
Structure superfactor	2.8^b^	3.1^a^	3.0^a^	2.8^b^
Limit Exposure	2.6^b^	2.9^a^	2.8^ab^	2.6^b^
Consistent feeding routines	3.0^b^	3.3^a^	3.1^ab^	3.0^b^
Control superfactor	1.9^a^	1.8^a^	1.5^b^	1.4^b^
Restriction	1.9^a^	1.9^a^	1.4^b^	1.7^ab^
Pressure to eat	1.9^a^	1.6^b^	1.3^c^	1.3^c^

^a,b,c^ Means sharing a common superscript are not significantly different at *p* < .05

Items scored on a 5-point Likert scale: 0 = never and 4 = always

## Discussion

Results reveal that the Structure-Control in Parent Feeding (SCPF) questionnaire, provides some support for Grolnick and Pomerantz Parental structure and control model [11], with the solution including 2 overarching superfactors, one for Structure and a second for Control, and 4 subscales that assess orthogonal aspects of parental control in feeding (i.e., restriction, pressure to eat) versus structure in feeding (i.e., limit exposure, consistent feeding routines). Following initial scale development, exploratory factor analysis revealed acceptable fit for the 4 factor model and second-order exploratory factor analysis provided evidence for the presence of two superfactors in this sample of low-income mothers with a toddler participating in the Women, Infants, and Children program.

The parent feeding literature tends to focus on evaluating parents’ use of control to manage children’s intake of palatable energy-dense foods [12]. As a result, it is well evidenced that controlling feeding practices such as restriction has been shown to be counterproductive, increasing the attraction towards the very foods that parents attempt to restrict [9, 10]. Experimental studies conducted in laboratory settings have demonstrated that allowing children unrestricted access to large portions of highly palatable, energy dense snack foods, results in some but not all children eating in the absence of hunger [10, 39–44]. Children who report that their parents to restrict access to these foods in the home tend to overeat those same foods when they are readily available [44]. As a result, parents are often left asking, “*If restricting access to foods that I don’t want my child to eat is counterproductive, then what am I supposed to do?*” This is an important question given the current obesogenic environment where children are exposed to energy-rich, palatable foods that are high in fat and sugar.

Grolnick and Pomerantz’s model of parental control posits that parents’ use of structure and control in parenting are two orthogonal constructs that can be disassociated and that have differing effects on child development. In this framework, “control” refers to parents’ use of power-based, intrusive strategies to manipulate children’s behaviors. Structure is conceptualized as orthogonal to control, and includes the rules and routines that parents use to organize the home environment, to provide a predictable environment. The broader literature on parenting reveals that parental coercive control negatively impacts children’s development of self-regulation, predicting lower delay of gratification in children [14, 15], while structure-based, limit setting practices promote child social and emotional regulation [11, 15]. This parenting model was used to guide the development of the SCPF questionnaire, a self-report instrument of both structure and control among a sample of low-income mothers of toddlers participating in WIC.

The 4-factor SCPF questionnaire focused on two broad categories: controlling feeding practices and structure-based feeding. Results indicated that 2 factor models for both Structure and Control provided acceptable fit to the data as well as adequate internal consistency for the SCPF questionnaire. In addition, consistent with theoretical framework, the SCPF demonstrated discriminant and convergent validity. As expected, the Structure superfactor and Structure subfactors were positively associated with a responsive feeding style based on the CFSQ and structure-oriented subscales from the CPFP questionnaire, and negatively associated with the control-oriented subscales. Because all of the measured variables were self-reported by mothers, these results are subject to reporting bias; however, collectively, these findings support the validity of the SCPF questionnaire and are consistent with past studies reporting positive correlations between use of controlling feeding practices (e.g. restriction and pressure to eat) and authoritarian and coercive approaches to parenting [45–47].

Although use of control in parenting has been associated with control in feeding in past work [45, 46], inconsistent findings have been reported as well [47–50]. In one study, only one-third of the sample was found to be authoritarian in feeding *and* parenting. It has been argued by Costanzo and Woody [51], and other researchers [12], that parents’ use of control is domain-specific and that parents may implement controlling feeding practices in response to children’s eating behaviors and weight gain, regardless of their general parenting approach. There currently are no data to indicate that structuring the home environment by setting limits, routines and expectations around when, when and how foods are made available, as assessed by the SCPF questionnaire, influences eating behavior and weight. There is also a lack of evidence regarding the optimal levels of structure and control in feeding, how these factors interact to influence eating behavior, and how parent and child characteristics may moderate these effects. The new SCPF questionnaire is designed as a tool to address some of these questions. Further, very little is currently known about parents’ motivations for using structure-based feeding practices.

Although not empirically tested, public health messages from the USDA Food and Nutrition Services recommend the “division of feeding responsibility” [52] to promote self-regulation. Although it is argued that the “division of feeding responsibility” alone, without structure-based feeding, may be enough to promote self-regulation and to prevent overeating in our obesogenic environment, the effects of “the division of feeding responsibility” are likely to be contingent on the “details”—the what and when of foods available in the home. Findings from recent work provide some evidence on this point. Rollins and colleagues [19] report that parents use of limit setting around children’s access to snack foods was protective of girls’ weight gain from ages 5 to 7, while coercive control predicted greater increases in disinhibited eating over the same period. In other research [20] parents’ use of controlling feeding practices (“How often are you firm about what your child should eat?”) was associated with greater snack intake in 4–11 year old children; in contrast, the opposite trend was present for structure-based (covert) feeding practices (“Avoid going to cafes or restaurants with your children which sell unhealthy foods?”).

## Conclusion

The SCPF questionnaire provides a validated, theory-based tool for assessing aspects of mothers’ structure in feeding and control in feeding. Results provide empirical support for characterizing differences among parental feeding practices in terms of aspects of structure (e.g., feeding routines, limit exposure) as well as coercive control (e.g., restriction and pressure to eat). This measure will be used to evaluate whether interventions focused on modifying mothers’ use of structure and control in feeding affect children’s dietary behaviors and weight status. Because the SCPF questionnaire was developed for use with a sample of WIC mothers and toddlers at elevated risk for obesity, additional research is needed to determine the extent to which this instrument is appropriate for diverse populations differing in demographics, and child characteristics.

## Abbreviations

BMI: Body mass index
CESD: Center for epidemiologic studies of depression scale
CFSQ: Caregiver’s feeding styles questionnaire
CPFP: Control in parent feeding practices questionnaire
EFA: Exploratory factor analysis
SCPF: Structure and control in parent feeding

## References

[1] Ogden CL, Carroll MD, Kit BK, Flegal KM (2012). Prevalence of obesity and trends in body mass index among US children and adolescents, 1999–2010. JAMA.

[2] Wang Y (2001). Cross-national comparison of childhood obesity: the epidemic and the relationship between obesity and socioeconomic status. Int J Epidemiol.

[3] Singh GK, Siahpush M, Kogan MD (2010). Rising social inequalities in US childhood obesity, 2003–2007. Ann Epidemiol.

[4] Skinner JD, Carruth BR, Wendy B, Ziegler PJ (2002). Children’s food preferences: a longitudinal analysis. J Am Diet Assoc.

[5] Hendy HM (2002). Effectiveness of trained peer models to encourage food acceptance in preschool children. Appetite.

[6] Young EM, Fors SW, Hayes DM (2004). Associations between perceived parent behaviors and middle school student fruit and vegetable consumption. J Nutr Educ Behav.

[7] Cullen KW, Baranowski T, Rittenberry L, Cosart C, Hebert D, de Moor C (2001). Child-reported family and peer influences on fruit, juice and vegetable consumption: reliability and validity of measures. Health Educ Res.

[8] Birch LL, Fisher JO (1998). Development of eating behaviors among children and adolescents. Pediatrics.

[9] Fisher JO, Birch LL (1999). Restricting access to palatable foods affects children’s behavioral response, food selection, and intake. Am J Clin Nutr.

[10] Rollins BY, Loken E, Savage JS, Birch LL (2014). Effects of restriction on children’s intake differ by child temperament, food reinforcement, and parent’s chronic use of restriction. Appetite.

[11] Grolnick WS, Pomerantz EM (2009). Issues and challenges in studying parental control: toward a new conceptualization. Child Dev Perspect.

[12] Rollins BY, Savage JS, Fisher JO, Birch LL (2016). Alternatives to restrictive feeding practices to promote self-regulation in childhood: a developmental perspective. Pediatr Obes.

[13] Vaughn AE, Ward DS, Fisher JO (2016). Fundamental constructs in food parenting practices: a content map to guide future research. Nutr Rev.

[14] Karreman A, van Tuijl C, van Aken MAG, Deković M (2006). Parenting and self-regulation in preschoolers: a meta-analysis. Infant Child Dev.

[15] Houck GM (2004). Maternal limit setting during toddlerhood, delay of gratification, and behavior problems at age five. Infant Mental Health J.

[16] Schlam TR, Wilson NL, Shoda Y, Mischel W, Ayduk O (2013). Preschoolers’ delay of gratification predicts their body mass 30 years later. J Pediatr.

[17] Baldwin AL (1955). Behavior and development in childhood.

[18] Symonds PM (1949). The dynamics of parent–child relationships.

[19] Rollins BY, Loken E, Savage JS, Birch LL (2014). Maternal controlling feeding practices and girls’ inhibitory control interact to predict changes in BMI and eating in the absence of hunger from 5 to 7 y. Am J Clin Nutr.

[20] Ogden J, Reynolds R, Smith A (2006). Expanding the concept of parental control: a role for overt and covert control in children’s snacking behaviour?. Appetite.

[21] Rodgers RF, Paxton SJ, McLean SA (2013). Do maternal body dissatisfaction and dietary restraint predict weight gain in young pre-school children? a 1-year follow-up study. Appetite.

[22] Campbell K, Andrianopoulos N, Hesketh K (2010). Parental use of restrictive feeding practices and child BMI z-score. A 3-year prospective cohort study. Appetite.

[23] Anzman-Frasca S, Stifter CA, Paul IM, Birch LL (2013). Infant temperament and maternal parenting self-efficacy predict child weight outcomes. Infant Behav Dev.

[24] Connell LE, Francis LA (2014). Positive parenting mitigates the effects of poor self-regulation on body mass index trajectories from ages 4–15 years. Health Psychol.

[25] Francis LA, Susman EJ (2009). Self-regulation and rapid weight gain in children from age 3 to 12 years. Arch Pediatr Adolesc Med.

[26] Vaughn AE, Tabak RG, Bryant MJ, Ward DS (2013). Measuring parent food practices: a systematic review of existing measures and examination of instruments. Int J Behav Nutr Phys Act.

[27] Musher-Eizenman D, Holub S (2007). Comprehensive feeding practices questionnaire: validation of a new measure of parental feeding practices. J Pediatr Psychol.

[28] Birch LL, Fisher JO, Grimm-Thomas K, Markey CN, Sawyer R, Johnson SL (2001). Confirmatory factor analysis of the child feeding questionnaire: a measure of parental attitudes, beliefs and practices about child feeding and obesity proneness. Appetite.

[29] Hurley KM, Black MM, Papas MA, Caulfield LE (2008). Maternal symptoms of stress, depression, and anxiety are related to nonresponsive feeding styles in a statewide sample of WIC participants. J Nutr.

[30] Sacco LM, Bentley ME, Carby-Shields K, Borja JB, Goldman BD (2007). Assessment of infant feeding styles among low-income African-American mothers: comparing reported and observed behaviors. Appetite.

[31] Thompson AL, Mendez MA, Borja JB, Adair LS, Zimmer CR, Bentley ME (2009). Development and validation of the infant feeding style questionnaire. Appetite.

[32] Murashima M, Hoerr SL, Hughes SO, Kaplowitz S (2011). Confirmatory factor analysis of a questionnaire measuring control in parental feeding practices in mothers of head start children. Appetite.

[33] Corsini N (2008). Exploring aspects of parental control over feeding: influences on children’s eating behaviour and weight.

[34] Hughes SO, Power TG, Orlet Fisher J, Mueller S, Nicklas TA (2005). Revisiting a neglected construct: parenting styles in a child-feeding context. Appetite.

[35] Radloff LS (1977). The CES-D scale: a self report depression scale for research in the general population. Appl Psychol Meas.

[36] Osborne J, Banjanovic E (2016). Exploratory factor analysis with SAS.

[37] Thompson B (2004). Exploratory and confirmatory factor analysis: understanding concepts and applications.

[38] Cronbach LJ (1951). Coefficient alpha and the internal structure of tests. Psychometrika.

[39] Remy E, Issanchou S, Chabanet C, Boggio V, Nicklaus S (2015). Impact of adiposity, age, sex and maternal feeding practices on eating in the absence of hunger and caloric compensation in preschool children. Int J Obes.

[40] Birch LL, Savage JS, Fisher JO (2015). Right sizing prevention. Food portion size effects on children’s eating and weight. Appetite.

[41] Savage JS, Fisher JO, Marini M, Birch LL (2012). Serving smaller age-appropriate entree portions to children aged 3–5 y increases fruit and vegetable intake and reduces energy density and energy intake at lunch. Am J Clin Nutr.

[42] Savage JS, Haisfield L, Fisher JO, Marini M, Birch LL (2012). Do children eat less at meals when allowed to serve themselves?. Am J Clin Nutr.

[43] Mooreville M, Davey A, Orloski A (2015). Individual differences in susceptibility to large portion sizes among obese and normal-weight children. Obesity.

[44] Fisher JO, Birch LL (2002). Eating in the absence of hunger and overweight in girls from 5 to 7 y of age. Am J Clin Nutr.

[45] Hubbs-Tait L, Kennedy TS, Page MC, Topham GL, Harrist AW (2008). Parental feeding practices predict authoritative, authoritarian, and permissive parenting styles. J Am Diet Assoc.

[46] Joyce JL, Zimmer-Gembeck MJ (2009). Parent feeding restriction and child weight. The mediating role of child disinhibited eating and the moderating role of the parenting context. Appetite.

[47] Hennessy E, Hughes SO, Goldberg JP, Hyatt RR, Economos CD (2010). Parent behavior and child weight status among a diverse group of underserved rural families. Appetite.

[48] Blissett J, Haycraft E (2008). Are parenting style and controlling feeding practices related?. Appetite.

[49] Johnson R, Welk G, Saint-Maurice PF, Ihmels M (2012). Parenting styles and home obesogenic environments. Int J Environ Res Public Health.

[50] Rodenburg G, Kremers SP, Oenema A, van de Mheen D (2013). Associations of parental feeding styles with child snacking behaviour and weight in the context of general parenting. Public Health Nutr Mar.

[51] Costanzo PR, Woody EZ (1985). Domain-specific parenting styles and their impact on the child’s development of particular deviance: the example of obesity proneness. J Soc Clin Psychol.

[52] United States Department of Agriculture: Food and Nutrition Services (2015). Maximizing the message: helping moms and kids make healthier food choices.

